# Minimally Invasive Resection of Occult Insulinomas—Experience from an ENETS Centre of Excellence and Review of the Literature

**DOI:** 10.3390/cancers17233857

**Published:** 2025-11-30

**Authors:** Alina S. Ritter, Feline Ockenga, Kira C. Steinkraus, Jelte Poppinga, Philipp H. von Kroge, Tania Amin, Fabrice Viol, Thorben W. Fründt, Felix Nickel, Thilo Hackert, Anna Nießen

**Affiliations:** 1 Department of General, Visceral and Thoracic Surgery, University Medical Center Hamburg-Eppendorf, 20246 Hamburg, Germany; a.ritter@uke.de (A.S.R.); f.ockenga@uke.de (F.O.); k.steinkraus@uke.de (K.C.S.); j.poppinga@uke.de (J.P.); p.von-kroge@uke.de (P.H.v.K.); f.nickel@uke.de (F.N.); t.hackert@uke.de (T.H.); 2 I. Department of Medicine, University Medical Center Hamburg-Eppendorf, 20246 Hamburg, Germany; t.amin@uke.de (T.A.); f.viol@uke.de (F.V.); t.fruendt@uke.de (T.W.F.)

**Keywords:** insulinoma, occult, neuroendocrine tumour, pNET, functionally active, minimally invasive surgery, pancreatic surgery

## Abstract

**Background/Objectives**: Insulinomas are rare insulin-secreting pancreatic neuroendocrine tumours (pNETs). Preoperative tumour localisation can usually be achieved by computed tomography (CT), magnetic resonance imaging, or positron emission tomography (PET)-CT. However, cross-sectional imaging can be negative, defining an insulinoma as occult and thus hampering surgical resection. **Methods**: All patients who underwent minimally invasive (MI) surgery for an insulinoma at the University Medical Center Hamburg-Eppendorf since 2017 were analysed. Clinicopathological parameters and diagnostic and operative approaches were assessed. A literature search of the MI resection of occult insulinomas was conducted. **Results**: Of eight patients with MI-resected insulinomas, two (25%) had negative preoperative imaging. Mean tumour size was 17.2 ± 13.3 mm. Patients underwent distal pancreatectomy (DP), enucleation, and pancreatic head resection (PHR) in 62.5% (5/8), 25.0% (2/8), and 12.5% (1/8) of cases, respectively. One patient had a major postoperative complication (Clavien–Dindo ≥ 3a). Twenty-four studies reporting on 140 occult insulinomas were identified. Occult insulinomas were more frequent in females, often located in the distal pancreas and G1-differentiated. Glucagon-Like Peptide-1 Receptor/PET-CT most frequently localised the conventionally non-visible insulinomas (positive in 67/76, 88.2%). Enucleation, DP, PHR and other resections were conducted in 47/94 (50.0%), 40/94 (42.6%), 4/94 (4.3%), and 3 (3.2%) of the reported cases. MI resection was reported in 10 of 19 (52.6%) specified resections. **Conclusions**: Insulinomas can be undetectable in cross-sectional and functional imaging. Surgical exploration with intraoperative ultrasound should be considered when clinical presentation and biochemical findings are highly suggestive for insulinoma. Minimally invasive and parenchyma sparing resection is feasible even for occult insulinomas and should always be considered.

## Simple Summary

Insulinomas are rare pancreatic neuroendocrine tumours that systemically secrete insulin, resulting in severe hypoglycaemia. In some cases, insulinoma cannot be localised through radiological imaging and are thus called occult insulinomas. Surgical resection remains the only cure for insulinomas and is preferably performed minimally invasively. All insulinomas that were resected minimally invasively at the University Medical Center Hamburg-Eppendorf between 2017 and 2025 were analysed. Two of eight insulinomas could not be localised in conventional imaging, but were still successfully and safely resected with keyhole surgery. In the available literature, the minimally invasive and parenchyma-sparing resection of occult insulinomas has likewise been reported as feasible. Intraoperative ultrasound can facilitate intraoperative tumour localisation. Occult insulinomas were more frequent in females and more often localised in the distal pancreas.

## 1. Introduction

Insulinomas are rare insulin-producing pancreatic neuroendocrine tumours (pNETs) with an incidence of 1–3 million/year, yet they are the most common cause of endogenous hyperinsulinaemic hypoglycaemia in adults [1]. While 85–90% of insulinomas are benign, aggressive insulinomas can occur [2]. The so-called “Whipple triad” consisting of (1) autonomic symptoms during fasting (e.g., sweating, tremor, confusion, seizure), (2) a low blood glucose level at the time of symptoms, and (3) relief of symptoms after normalisation of blood glucose is the diagnostic hallmark of insulinomas [1].

Following biochemical suspicion of an insulinoma, localisation is crucial. Most insulinomas are located within the pancreas; however, rare ectopic manifestations in the lung, small intestine, or spleen have been described [3,4]. Additionally, approximately 30% of insulinomas are less than 1 cm in size and 10% occur multifocally [1,3]. Multiple diagnostic approaches have been established in order to localise pancreatic insulinomas: transabdominal ultrasound, computed tomography (CT), and magnetic resonance imaging (MRI) are widely available, non-invasive imaging strategies [5]. However, not all insulinomas are visible on cross-sectional imaging and are thus considered occult insulinomas [3,6]. This can delay therapy significantly and can expose patients to life-threating hypoglycaemia.

Besides cross-sectional imaging, invasive techniques such as endoscopic ultrasound (EUS) and fine needle aspiration (FNA) can facilitate diagnostic confirmation in unclear cases, but they are more invasive and observer-dependent [7,8]. Another invasive approach to localise insulinoma is selective pancreatic angiography and calcium stimulation catheterisation (SACST), but it only identifies the vascular bed and not the tumour itself, and can therefore be affected by variants in vascular anatomy or overlapping vascular territories [9,10].

Nowadays, Glucagon-Like Peptide-1 Receptor positron emission tomography (GLP-1R PET/CT) after injection of 68Ga-DOTA-exedin-4 offers a non-invasive method to detect benign insulinomas, exploiting the high expression density of GLP-1 receptors on insulinoma cells; however, this technique is not universally available [11,12]. The more widely accessible ^68^Ga-DOTATATE/PET-CT, marking the somatostatin receptor, offers lower sensitivity in the detection of insulinomas [5].

Following diagnostic work-up, the standard of care is the resection of insulinomas, which usually provides cure [13]. Preoperatively, the precise localisation of insulinomas is essential in order to plan the adequate resection technique while simultaneously preserving healthy pancreas parenchyma in order to prevent the development of postoperative diabetes [14]. Nowadays, this can routinely be carried out minimally invasively if the insulinoma was localised preoperatively [3,15,16]. However, it is unknown whether GLP-1R PET/CT enables a minimally invasive resection more frequently. If the localisation of an insulinoma is not successful, blind resection of the pancreas is not recommended as this could result in unsuccessful treatment of the patient and the unnecessary sacrifice of healthy pancreatic tissue [3,13]. However, this also poses a risk of persistence of the potentially life-threatening hypoglycaemic episodes.

Thus, a retrospective analysis of patients with insulinomas who underwent minimally invasive resection was performed, identifying those in whom this approach was successful despite negative preoperative cross-sectional imaging. The available literature on minimally invasive surgery for occult insulinomas is presented in this paper.

## 2. Materials and Methods

### 2.1. Patient Selection

A retrospective analysis of all patients presenting with biochemically proven pancreatic insulinoma between January 2017 and May 2025 at the Centre for Neuroendocrine Tumours of the University Medical Center Hamburg-Eppendorf, Hamburg, Germany, was conducted. The institution is certified as a Center of Excellence (CoE) by the European Neuroendocrine Tumor Society (ENETS). This time span was chosen as GLP-1R PET/CT and “DaVinci Xi” system (Intuitive, Sunnyvale, CA, USA) both became available in 2017. The inclusion criteria included a pathological fasting test, a negative preoperative CT and/or MRI scan (depending on the retrospectively available imaging), a minimally invasive surgical approach, and a histologically proven insulin-positive neuroendocrine tumour. The exclusion criteria included functionally inactive pNETs and visibility on preoperative CT or MRI. This study was approved by the local ethics committee (Ethikkommission der Ärztekammer Hamburg, 2025-101580-BO-ff, 24 September 2025). Informed consent was waived by the ethics committee due to the retrospective nature of the study and in accordance with the local legislation (HmbKHG §12). Written informed consent was obtained from the patient whose imaging is published in this paper.

### 2.2. Clinical Data

**Figure 1:**
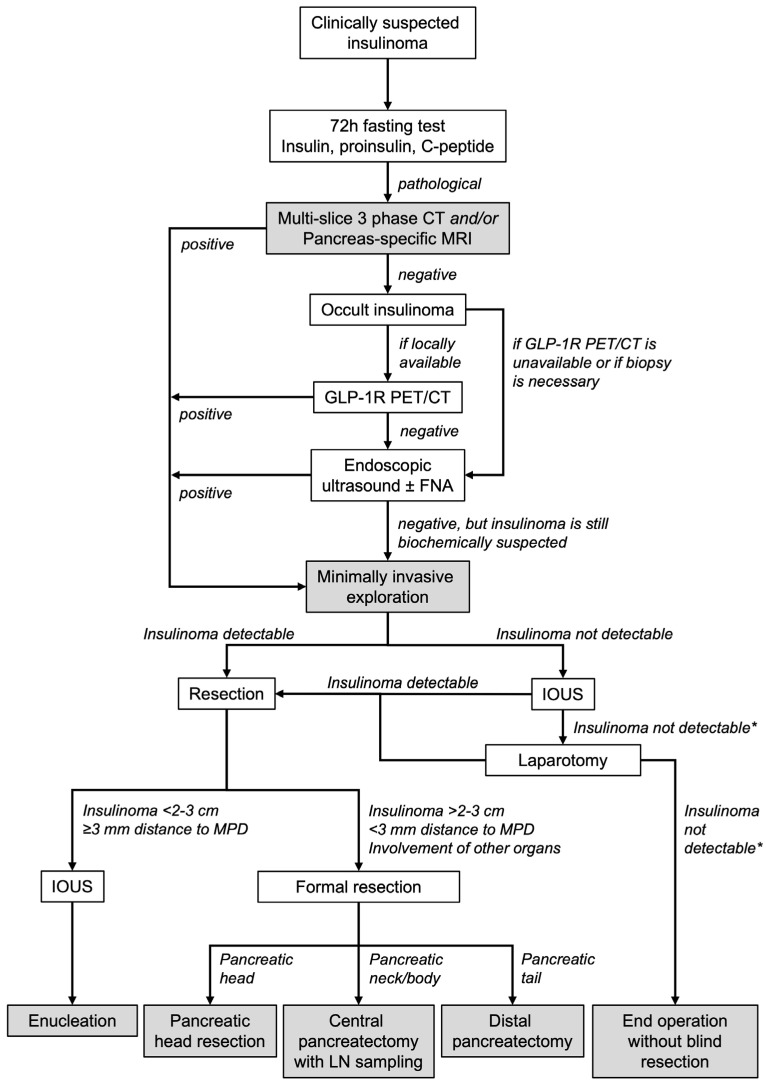
Preoperative diagnostic work-up and operative decision tree in patients with insulinoma. All insulinoma patients should be discussed in a multidisciplinary team, especially in cases of negative imaging. In cases of negative EUS without prior GLP-1R/PET-CT, this may also be conducted afterwards if available. * Intraoperative administration of indocyanine green might additionally aid insulinoma detection; however, data on this is scarce. cm: centimetre, CT: computed tomography, FNA: fine needle aspiration, GLP-1R PET/CT: Glucagon-Like Peptide-1 Receptor positron emission tomography, IOUS: intraoperative ultrasound, LN: lymph node sampling, mm: millimetre, MPD: main pancreatic duct, MRI: magnetic resonance imaging.

Patients were identified from the institution’s database and data were extracted from the digital electronic patient files. The preoperative diagnostic work-up and operative decision tree are summarised in Figure 1. If an insulinoma was suspected clinically, a 72 h fasting test was conducted to provide biochemical proof of an insulinoma. In case of a pathological fasting test, insulinoma localisation was sought with multi-slice 3-phase CT or pancreas-specific MRI. In ambiguous cases, EUS and FNA were carried out. If these were all negative but an insulinoma remained clinically and biochemically likely, since 2017, a GLP-1R PET/CT was conducted after case discussion in a multidisciplinary team (MDT) with experts in the treatment of neuroendocrine tumours (NETs).

Occult insulinoma was defined by negative preoperative CT and/or MRI if both imaging types were available in patients with biochemically suspected (i.e., pathological 72 h fasting test) insulinoma. Since GLP-1R PET/CT is not routinely available and as EUS is an invasive, investigator-dependent technique, this was not applied in the definition of an occult insulinoma.

The operative strategy was decided on by an experienced pancreatic surgeon based on the preoperative imaging as well as the individual patient’s clinical and surgical history. A laparoscopic or robot-assisted approach was the local standard of care whenever feasible. The decision regarding enucleation or formal resection was made intraoperatively in accordance with the ENETS guidelines [13]: enucleation was conducted in localized tumours < 2–3 cm and if the distance to the main pancreatic duct was ≥3 mm; otherwise, formal resection depending on the tumour localisation within the pancreas (pancreatic head: pancreatic head resection (PHR), pancreatic neck/body: central pancreatectomy with lymph node sampling, pancreatic tail: distal pancreatectomy (DP)) was performed. The decision to carry out an intraoperative ultrasound (IOUS) was made by the pancreatic surgeon and was based on the detectability of the insulinoma. However, after 2022, IOUS was routinely performed if an enucleation was performed. Postoperative complications were graded according to Clavien–Dindo classification (CDC) [17]. A complication with a CDC ≥ 3a was considered a major complication. For pancreatic surgery-specific complications, the definitions of the International Study Group for Pancreatic Surgery (ISGPS) for postoperative pancreatic fistula (POPF) [18], delayed gastric emptying (DGE) [19], post-pancreatectomy haemorrhage [20], and bile leak [21] were applied. Histopathological evaluation of FNAs and resection specimens was conducted by the institution’s pathology department based on the current World Health Organization classification and the 8th edition of the TNM classification [22,23].

### 2.3. Literature Search

A systematic literature search was conducted in accordance with the Preferred Reporting Items for Systematic Reviews and Meta-Analyses (PRISMA) guidelines [24]. The National Library of Medicine’s PubMed database (Bethesda, MD, USA) was searched for articles published between May 2015 and May 2025 using the Medical Subject Heading (MeSH) terms (“Insulinoma”[MeSH] OR “Insulinoma” OR “Insulinomas” OR “pancreatic neuroendocrine tumor” OR “pNET”) AND (“Occult” OR “Non-localized” OR “Unlocalized” OR “Hidden”) AND (“Diagnosis”[MeSH] OR “Diagnosis” OR “Detection” OR “Imaging”). An additional database search in the Web of Science (Clarivate Analytics, Philadelphia, PA, USA) using the topics TS = (insulinoma OR insulinomas OR “pancreatic neuroendocrine tumor” OR “pNET”) AND TS = (“occult” OR “hidden” OR “non-localized” OR “unlocalized”) AND TS = (diagnosis OR detection OR imaging) was conducted. Databases were last consulted on 26 May 2025.

Publications and case reports were included if they reported on insulinomas that were not visible in CT and MRI imaging or negative in CT or MRI if only one of these was conducted. The exclusion criteria were overt insulinomas (visible in MRI or CT), reviews, studies in animals, or publications not available in English or German. Two independent reviewers (A.S.R and F.O.) screened the abstracts for eligibility and extracted the data. Discrepancies were resolved by consensus, and in case of disagreement, a third reviewer was consulted (K.C.S.). The following information was extracted: first author, year of publication, number of reported patients, gender, presence of genetic syndrome, conducted preoperative diagnostics, surgical resection, operative approach, extent of resection, conduction of IOUS, postoperative complications, tumour size, histology, and insulinoma localisation within the pancreas. Data was independently tabulated, summarised using a predefined data collection form, and displayed descriptively. Missing and unspecified values were excluded from the data summary.

Patients identified from the authors’ institutional database were combined with the extracted data (“Hamburg cohort”).

### 2.4. Data Analysis

All values are given as absolute numbers and percentages (*n*/%) with mean and standard deviation (SD) or median with interquartile range (IQR) for continuous variables, as indicated. The analysis was conducted with GraphPad Prism^®^ 10.4.2 (GraphPad Software, Boston, MA, USA).

## 3. Results

### 3.1. Hamburg Cohort of Occult Insulinomas

Between January 2017 and May 2025, eight patients with biochemically proven insulinomas underwent minimally invasive resection at the University Medical Center Hamburg-Eppendorf, Hamburg, Germany. Their clinical, diagnostic, and operative details are summarised in Table 1.

**Figure 2:**
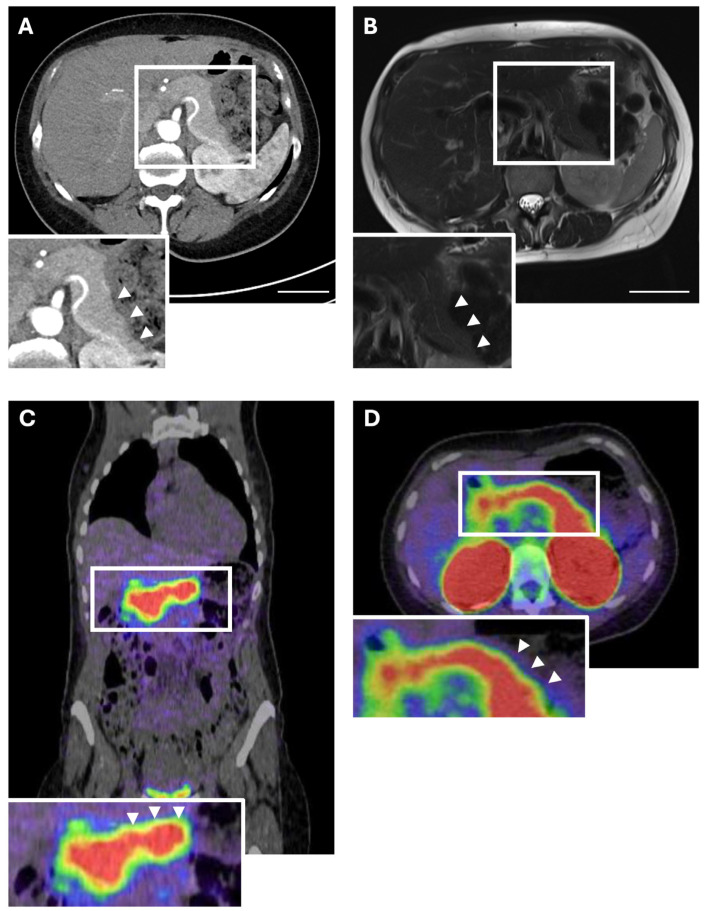
Preoperative imaging of an occult insulinoma. (**A**) Computed tomography and (**B**) T2-weighted magnetic resonance imaging of a patient with an occult insulinoma showing no intrapancreatic lesion. (**C**) Coronal view of a GLP-1R PET/CT of the same patient with an occult insulinoma showing no pathological focal tracer enhancement in the pancreas. (**D**) Axial view of the same GLP-1R PET/CT of the same patient with an occult insulinoma showing no pathological focal tracer enhancement in the pancreas. Scale bars represent 5 cm. Region of interest (white square) is magnified in the respective images below (**A**: 1.4-fold magnification, **B**: 1.3-fold magnification, **C**: 1.7-fold magnification, **D**: 1.4-fold magnification). White arrowheads mark the pancreatic tail, where an occult insulinoma was later detected histologically.

The median age of the “Hamburg cohort” was 50.5 years (IQR 47.25–65.25 years) with an equal gender distribution (4:4, 50%:50%). No patient had multiple endocrine neoplasia (MEN) type 1 syndrome. All patients received either preoperative CT or MRI. In ambiguous cases, both modalities were combined and EUS was additionally conducted. Of all patients, preoperative cross-sectional imaging with MRI and CT revealed no pancreatic lesion in two cases (2/8, 25.0%). One patient was male aged 68, whereas the other one was female aged 51, at the time of diagnosis. Both patients underwent EUS upon negative imaging, which likewise showed no pancreatic lesion. In one of these patients, repetition of EUS after 12 months displayed a pNET, however, cross-sectional imaging remained negative. After case discussion in the MDT, both patients received a GLP-1R PET/CT due to the persistence of hypoglycaemic episodes. For one patient, this revealed a positive lesion, whereas it remained negative for the other (Figure 2).

All patients underwent minimally invasive exploration. The operation was converted to open surgery in one laparoscopic case—to enable safe enucleation that, due to the proximity of the tumour to the main pancreatic duct, would otherwise not have been possible—and in one robotic case, as sufficient muscle relaxation in order to maintain capnoperitoneum was not achievable. No conversions occurred after 2018 and no conversion was necessary to localise the insulinoma. The majority of patients underwent distal pancreatectomy (5/8, 62.5%), followed by enucleation (2/8, 25%) and PHR (1/8, 12.5%). Reasons for formal resections included tumour size > 3 cm (1/6, 16.7%), proximity to the main pancreatic duct (4/6, 66.7%), and involvement of the splenic hilum (1/6, 16.7%).

**Figure 3:**
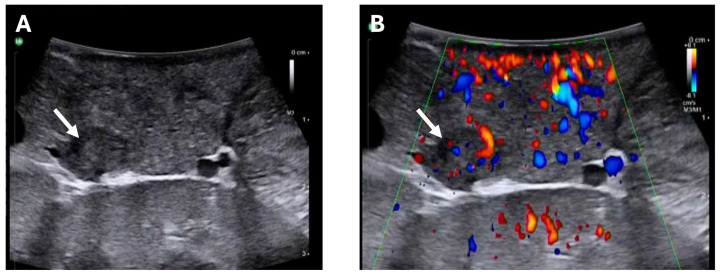
Intraoperative robot-assisted ultrasound of occult insulinoma. (**A**) B-mode and (**B**) colour-coded doppler sonography of the same patient with occult insulinoma localising an intrapancreatic, hypervascularised lesion (arrows).

Concerning the two patients with occult insulinoma specifically, both underwent minimally invasive exploration. In both cases, based on intraoperative findings and aided with IOUS in one case (Figure 3), spleen-preserving distal pancreatectomy was conducted. In neither case was conversion to open surgery necessary. In one of these patients, a drainage was placed intraoperatively.

The majority of patients had a G2 tumour (6/8, 75.0%). One patient had a malignant tumour with a pT3 pN1 pM1 stage. All other patients were staged pT1 pN0 cM0. The mean tumour size was 17.2 ± 13.3 (SD) mm. Specifically, for both patients with negative preoperative imaging upon histopathological evaluation of the resected specimens, a G2-graded pNET staining positive for insulin was found. The mean tumour size of those was 13.0 mm ± 4.2 (SD) mm.

The postoperative course remained uneventful in most patients. The median length of postoperative hospital stay was 8.5 days (IQR: 6.5–18.25) and five patients participated in the local “Enhanced Recovery After Surgery (ERAS)” pathway [25]. One patient developed DGE grade B and underwent endoscopic pylorus dilatation after robotic pylorus preserving PHR [19]. Apart from that, no major complications (CDC ≥ 3a) were observed. Specifically, no patient developed POPF [18]. No postoperative 30-day or 90-day mortality was observed. No patient was readmitted to the hospital within 30 days. Postoperative hypoglycaemia ceased in all seven patients with benign disease and did not reoccur in the long-term follow up. This specifically included the patients with occult insulinomas, indicating that the operative treatment was successful.

The median follow up was 15.5 months (IQR: 2.0–58.25 months). The patient with metastatic, malignant insulinoma died of disease 17 months postoperatively. Two patients (2/8, 25.0%) required oral pancreatic enzyme replacement therapy. No patient with benign insulinoma developed endocrine pancreatic insufficiency or local recurrence during follow up.

Based on the herewith presented case series, routine minimally invasive resection of insulinomas can be encouraged. Importantly, even in patients with negative cross-sectional imaging despite the use of GLP-1R PET/CT and ambiguous preoperative tumour localisation, minimally invasive resection with IOUS is feasible, effective, and safe.

### 3.2. Systematic Review on Occult Insulinomas

**Figure 4:**
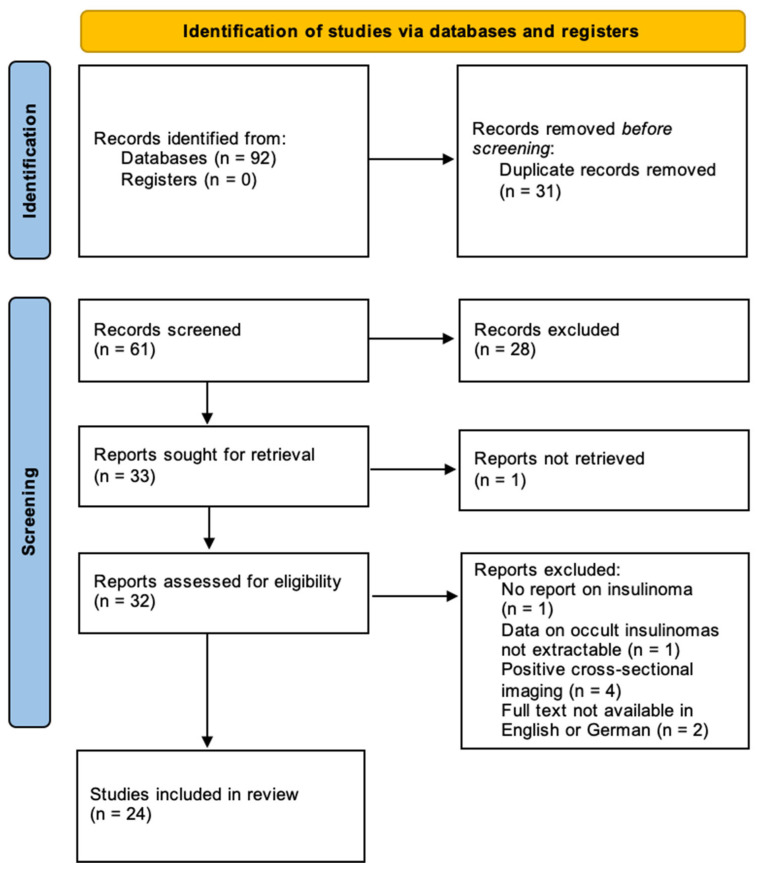
Study selection flowchart according to PRISMA recommendations.

The available literature on occult insulinomas was systematically searched to further evaluate the feasibility and safety of minimally invasive surgery in occult tumours. The PRISMA flow chart is displayed in Figure 4. The review was not registered.

After initial screening, 32 articles were considered eligible for assessment [9,10,12,26,27,28,29,30,31,32,33,34,35,36,37,38,39,40,41,42,43,44,45,46,47,48,49,50,51,52,53,54]. Of those, eight articles were excluded as they did not report on insulinoma (*n* = 1), data on occult insulinoma was not exclusively extractable (*n* = 1), cross-sectional imaging with MRI and/or CT was positive (*n* = 4), or full text was not available in English or German (*n* = 2). Finally, 24 studies reporting on 140 patients were identified [9,10,12,26,27,28,29,30,31,34,35,36,38,40,41,42,45,46,47,48,49,50,51,53]. Combined with the two occult insulinomas from the “Hamburg cohort”, a total of 142 occult insulinoma patients were analysed. The analysis was limited to a descriptive report of the literature as most studies were case reports and data were inconsistently reported, rendering a quantitative meta-analysis unfeasible. The baseline characteristics are summarised in Table 2.

**Figure 5:**
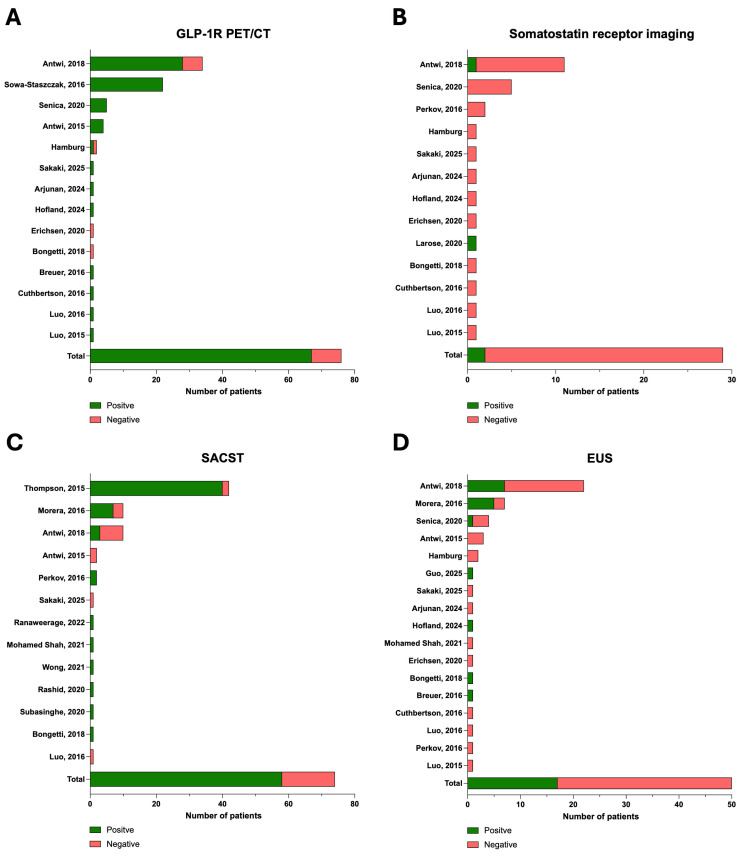
Preoperative imaging performed for occult insulinoma in the studies identified [9,10,12,26,27,28,29,30,31,34,35,36,38,40,41,42,45,46,47,48,49,50,51,53]. (**A**) Number of conducted Glucagon-Like Peptide-1 Receptor positron emission tomography (GLP-1R PET/CTs), (**B**) somatostatin receptor imagingss, (**C**) selective pancreatic angiography calcium stimulation catheterisations (SACST) and (**D**) endoscopic ultrasounds in the reported studies. Only studies reporting on the respective imaging modality are displayed. True positive occult insulinomas are displayed in green, whereas false negative cases are displayed in red.

The median age was 48.0 years (IQR 35.0–82.0 years) with a gender ratio of 34:59 (36.6%:63.4%). Of those, seven (6.7%) patients had known Multiple endocrine neoplasia (MEN)-1 syndrome. Due to the small number of MEN patients, these were pooled with sporadic cases. Preoperative CT and MRI were conducted in 72/138 (52.2%) and 118/136 (86.8%) cases, respectively (Table 3). Somatostatin receptor imaging was positive in 2 of 27 (7.4%) conducted cases. GLP-1R PET/CT was reported in 76/96 (79.2%) patients and positive for 67/76 (88.2%) of those (Table 3 and Figure 5). Additional EUS was reported on in 50/96 (52.1%) of cases and positive in 17/50 (34.0%) patients. A Selective Arterial Calcium Stimulation Test (SACST) was able to localise the occult insulinoma in 58 of 74 (78.4%) conducted cases (Table 3).

**Figure 6:**
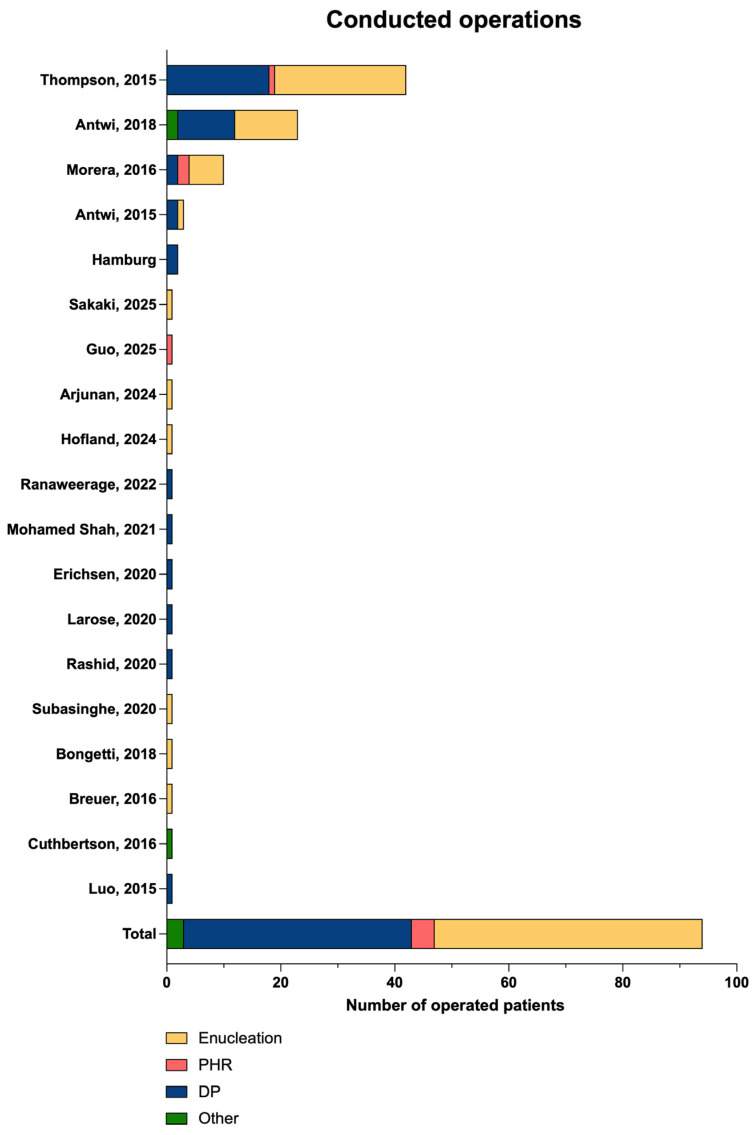
Overview of the conducted resection techniques for the respective studies reporting enucleation (yellow), pancreatic head resection (PHR, red), distal pancreatectomy (DP, blue), and other resection techniques (green) [extracted from [9,10,12,26,27,28,29,30,31,34,35,36,38,40,41,42,45,46,47,48,49,50,51,53].

The insulinoma was resected in 125 of 142 (88.0%) patients (Table 4). The applied resection technique was reported in 94 cases: of those, the most common type of operation was enucleation (47/94, 50.0%), followed by distal pancreatectomy (40/94, 42.6%) and pancreatic head resection (4/94, 4.3%; Figure 6).

Ten (52.6%) operations in total were performed minimally invasively. However, the approach was only specified in 19 of 125 operations (Table 4). Intraoperative ultrasound was reported to have been used in 53 cases (Table 4). Postoperative major complications were reported in 2 of 83 patients (2.4%), resulting in the death of one patient at an undefined time point (Table 4). Details on length of hospital stay, readmission, POPF rate, and DGE were not sufficiently reported for extraction.

The mean tumour size was 14.2 ± 4.5 (SD) mm. Most tumours were graded as G1 (44/56, 78.6%) and G2 (10/56, 17.9%), respectively. Occult insulinomas were most commonly located in the pancreatic body and tail (45/62, 72.6%), whereas 17 of 62 (27.4%) were found in the pancreatic head (Table 5).

Due to the often small size of insulinomas at the detection limits of conventional CT and MRI, endocrinologists and surgeons need to be aware of the possibility of occult insulinomas. In cases with negative conventional imaging but persisting hypoglycaemic episodes and pathological fasting test, GLP-1R PET/CT should be conducted if available as a next step, but can nevertheless yield false negative results. Even in cases of negative preoperative imaging, including negative GLP-1R PET/CT, the available literature suggests that minimally invasive exploration with endoscopic ultrasound is feasible and safe if patients display persisting symptoms indicative of an insulinoma. Parenchyma-sparing enucleation can frequently be conducted despite preoperative negative cross-sectional imaging.

## 4. Discussion

Recurring episodes of severe hypoglycaemia are the main characteristic of insulinomas initiating further diagnostic work-up. However, due to autonomic symptoms such as seizures, insulinomas can initially be misdiagnosed as epilepsy or neuropsychiatric disorders and thus delay accurate diagnosis and treatment [55]. The mean time to diagnosis for insulinoma patients is approximately 3.8 years, even for patients with overt insulinomas [56,57]. Negative imaging can further prolong this delay in diagnosis, exposing the patients to potentially life-threatening hypoglycaemic episodes in the meantime. Based on epidemiological data, the incidence is highest in the fifth decade, and females are more frequently affected than males [3]. Similarly, occult insulinomas are reported more frequently in females.

Biochemical diagnosis with the 72 h fasting test remains the gold standard for diagnosing insulinoma. More than 95% of cases can be detected with this diagnostic tool [1]. Localisation of the insulinoma is the next step in order to plan surgical resection. However, due to the frequently small size of benign insulinomas of 1–2 cm, resolution limits of CT and MRI scans as well as motion artifacts due to bowel movement, respiration, or cardiac pulsation can impact their detectability [3,5,13]. Sensitivity for insulinoma localisation ranges between 44 and 74% for CT and 56 and 90% for MRI imaging, respectively [5]. Endoscopic ultrasound has a high sensitivity of 70–100% and can detect lesions of 5 mm, but is operator-dependent, requires sedation, and is more invasive [3,5]. Additionally, it is less sensitive for the detection of lesions located in the pancreatic tail compared to those in the pancreatic head, where occult insulinomas are less frequent [3]. Yet, the possibility to conduct a fine needle aspiration and thus enable a preoperative histological analysis of a pancreatic tumour is a clear advantage of endoscopic ultrasound [3]. If insulinoma is clinically suspected, multiple imaging tools as well as endoscopic ultrasound should be combined and—as appropriate—repeated in order to increase sensitivity and confirm the suspected diagnosis.

Recently, GLP-1R PET/CT has been developed as an additional non-invasive diagnostic tool to detect insulinomas, exploiting the high expression density of GLP-1 receptors in benign insulinomas [11,58]. Its sensitivity is reported to be 94–98% [5]. GLP-1R PET/CT is less sensitive in malignant insulinoma, as they lack GLP-1 receptor overexpression [59]. However, this imaging technique is not widely available, is expensive to conduct, and contains an inevitable radiation burden [60]. Based on the above-mentioned sensitivities of these diagnostic tools, the probability of negative imaging with all three modalities ranges between 0.05 and 1.74% [5]. In the identified studies, GLP-1R PET/CT most frequently localised the insulinoma in cases of negative conventional imaging and should therefore be conducted as a next step—if locally available—in cases with negative MRI and CT but biochemically suspected insulinoma, as Somatostatin receptor imaging (SRI) and EUS show higher false negative results in the reported literature (Figure 5). Thus, one can hypothesise that GLP-1R PET/CT will reduce the number of occult insulinomas and can consecutively increase the rate of successful minimally invasive operations of occult insulinomas even further in the future. Due to incompletely reported datasets, no direct correlation could be shown here. Nevertheless, based on the author’s experience even in cases of a false negative GLP-1R PET/CT, minimally invasive exploration with IOUS is still feasible.

In cases of unclear preoperative imaging, potential differential diagnoses include adult nesidioblastosis—a diffuse proliferation of beta cells in the islets of Langerhans—as well as multiple or ectopic insulinoma, which would change the operative approach: while parenchyma-sparing pancreatic resection is possible in benign insulinoma, nesidioblastosis would require near total pancreatectomy, whereas multiple or ectopic insulinomas would need to be resected depending on their localisation [3,61]. In contrast to insulinomas, cross-sectional imaging is usually negative in adult nesidioblastosis; however, a diffuse increase in GLP-1R PET/CT can be detected [62].

If preoperative localisation remains doubtful, based on the available literature, minimally invasive exploration can still be performed if patients persistently show hypoglycaemic episodes and if the 72 h fasting test remains pathological. While the referral of insulinoma patients to an ENETS CoE and discussion in an MDT of NET experts is advisable in any case, it should be obligatory in patients with occult insulinoma and prior to operative exploration despite negative imaging. Intraoperative localisation with IOUS is an established technique that can even be applied when using a laparoscopic or robotic approach [3]. The available literature suggests that occult insulinomas are more frequent in the pancreatic body and tail. In case a suspected insulinoma cannot be found intraoperatively, it is recommended to end the operation and not perform blind resections [3]. Intraoperative frozen sections can support intraoperative tumour localisation and ensure complete resection. Promising novel localisation tools, such as intraoperative Indocyanine green angiography, might further facilitate insulinoma localisation in the future [63]. Once localised, a parenchyma-sparing resection, i.e., enucleation, is feasible for insulinomas and small non-functioning pNETs [3,64].

In recent years, minimally invasive surgery has been increasingly used for the resection of insulinomas, with parenchyma-sparing resections being the most frequently performed procedures [3,15,65]. Specifically, robotic pancreatic surgery has become the standard of care in many centres and is a safe approach for occult as well as overt insulinoma [66,67,68,69,70]. Regarding oncological radicality, robotic resections are not inferior to laparoscopic or open approaches, even for malignant insulinoma [69,70,71,72]. Nevertheless, most studies do not specifically focus on neuroendocrine tumours, but rather include all forms of pancreatic neoplasms. A disadvantage of robotic surgery thus far is the lack of haptic feedback and the fact that intraoperative tumour localisation by palpation is not possible [3]. However, IOUS, which has a mean sensitivity of 90%, can nevertheless be conducted and therefore enables parenchyma-sparing resections [3]. Thus, whenever possible and available, robotic pancreatic resections of insulinomas with IOUS can be encouraged even if preoperative tumour localisation is negative. After complete resection, the prognosis of benign insulinoma is very good with recurrence rates of < 10% [3,15].

In summary, based on the local clinical experience and the review of the available literature, the following “practice points” are considered the key clinical implications in the treatment of patients with occult insulinomas:

- Insulinomas can be negative in conventional MRI and CT. This is most frequent in female patients and in insulinomas of the pancreatic body and tail.
- If an insulinoma is clinically suspected (i.e., recurring hypoglycaemia and pathological fasting test) despite negative imaging, GLP-1R PET/CT should be conducted as an additional diagnostic tool if available.
- In case an insulinoma remains clinically suspected despite negative conventional imaging, EUS, and GLP-1R PET/CT, minimally invasive exploration with intraoperative ultrasound is feasible and safe.
- Parenchyma-sparing resections can be achieved even in cases of negative preoperative imaging.

Of note, in patients who are considered unfit for surgical resection, radiofrequency ablation of insulinomas has been introduced as an alternative treatment option. Nevertheless, pre-interventional tumour localisation with EUS is likewise vital [73].

Limitations of this study include the retrospective analysis and small sample size of the local collective due to the rarity of the disease. To increase the sample size, a systematic literature review was added. However, this bares limitations due to the heterogeneity of the reported studies. Additionally, not all assessed details were reported in the analysed studies, resulting in incomplete datasets. Data on the postoperative course were frequently not reported in detail, and thus postoperative complication rates might be underestimated.

## 5. Conclusions

In conclusion, insulinomas are rare neuroendocrine tumours that cause persistent severe hypoglycaemia. If an insulinoma is suspected, precise localisation should be established. In cases of negative imaging, multiple modalities should be combined. GLP-1R PET/CT has the highest sensitivity, but false negative results are nevertheless possible. Occult insulinomas are more frequent in females, small in size, and located in the distal pancreas. Minimally invasive, parenchyma-sparing surgery with intraoperative ultrasound can be feasible, even for patients with occult insulinomas. Complete resection of occult insulinomas usually results in cessation of symptoms. Patients should be treated in specialised centres with multidisciplinary care.

## Figures and Tables

**Table 1:** Clinical, diagnostic, operative, and histopathological details of patients undergoing minimally invasive resection of insulinomas between 2017 and 2025.

	Hamburg Cohort (*n* = 8)	
	*n*/All	IQR/%/SD
Median age [years]	50.5	47.25–65.25 (IQR)
Gender	4:4	50.0%:50.0%
**Preoperative diagnostics**		
Preoperative CT (positive/conducted)	3/5	60.0%
Preoperative MRI (positive/conducted)	5/8	62.5%
Preoperative EUS (positive/conducted)	3/5	60.0%
Preoperative SRI (positive/conducted)	1/4	25.0%
Preoperative GLP-1R PET/CT (positive/conducted)	1/2	50.0%
**Operative details**		
Insulinoma localisation	Head: 2/8	25.0%
	Body/Tail: 6/8	75.0%
Approach	Laparoscopic: 3/8	37.5%
	Robotic: 5/8	62.5%
Conversion rate	Laparoscopic: 1/3	33.3%
	Robotic: 1/5	20.0%
Operation	Enucleation: 2/8	25.0%
	PHR: 1/8	12.5%
	DP:5/8	62.5%
Postoperative drain use	Yes: 7/8	87.5%
	No: 1/8	12.5%
Postoperative major complication (CDC ≥ 3a)	1/8	12.5%
Median length of stay [days]	8.5	6.5–18.25 (IQR)
**Histopathological** **characteristics**		
Mean tumour size [mm]	17.2	13.3 (SD)
Tumour grading	G1: 2/8	25.0%
	G2: 6/8	75.0%
pT stage ^1^	pT1: 7/8	87.5%
	pT2: 0/8	0%
	pT3: 1/8	12.5%
	pT4: 0/8	0%
pN stage ^1^	pN0: 7/8	87.5%
	pN1: 1/8	12.5%
c/pM stage ^1^	c/pM0: 7/8	87.5%
	c/pM1: 1/8	12.5%
R stage ^1^	R0: 5/8	62.5%
	R1: 2/8	25.0%
	Rx: 1/8	12.5%

^1^ according to TNM 8th edition, CDC: Clavien–Dindo classification, CT: computed tomography, DP: distal pancreatectomy, EUS: endoscopic ultrasound, GLP-1R PET/CT: Glucagon-Like Peptide-1 Receptor positron emission tomography, IQR: interquartile range, mm: millimetre, MRI: magnetic resonance imaging, PHR: pancreatic head resection, SD: standard deviation, SRI: somatostatin receptor imaging.

**Table 2:** Clinical characteristics of patients with occult insulinomas extracted from studies.

Study	Number of Patients *n*	Male/Female (%) *n*:*n* (%:%)	Median Age (IQR) [Years]	MEN1-Patients *n* (%)
Hamburg cohort	2	1:1 (50.0:50.0)	59.5 (51–68)	0
Antwi, 2015 [26]	4	2:2 (50.0:50.0)	55.5 (41.3–63.8)	0
Antwi, 2018 [12]	34	*n*/A	N/A (N/A)	0
Arjunan, 2024 [27]	1	0:1 (0:100)	35 (N/A)	0
Bongetti, 2018 [28]	1	0:1 (0:100)	82 (N/A)	0
Breuer, 2016 [29]	1	0:1 (0:100)	64 (N/A)	0
Cuthbertson, 2016 [30]	1	0:1 (0:100)	49 (N/A)	0
Erichsen, 2020 [31]	1	0:1 (0:100)	14 (N/A)	1 (100)
Guo, 2025 [34]	1	0:1 (0:100)	44 (N/A)	0
Hofland, 2024 [35]	1	1:0 (100:0)	45 (N/A)	0
Larose, 2020 [36]	1	1:0 (100:0)	37 (N/A)	0
Luo, 2015 [40]	1	1:0 (100:0)	52 (N/A)	0
Luo, 2016 [38]	1	0:1 (0:100)	61 (N/A)	0
Mishra, 2022 [41]	4	N/A	N/A (N/A)	N/A
Mohamed Shah, 2021 [42]	1	1:0 (100:0)	33 (N/A)	0
Morera, 2016 [9]	11	N/A	N/A (N/A)	N/A
Perkov, 2016 [45]	2	0:2 (0:100)	33.5 (30–37)	1 (50.0)
Ranaweerage, 2022 [46]	1	0:1 (0:100)	23 (N/A)	1 (100)
Rashid, 2020 [47]	1	1:0 (100:0)	40 (N/A)	0
Sakaki, 2025 [48]	1	0:1 (0:100)	67 (N/A)	0
Senica, 2020 [49]	5	0:5 (0:100)	57.0 (41.5–68.5)	0
Sowa-Staszczak, 2016 [50]	22	10:12 (45.5:54.5)	48 (34–60.5)	N/A
Subasinghe, 2020 [51]	1	1:0 (100:0)	39 (N/A)	0
Thompson, 2015 [10]	42	15:27 (45.7:64.3)	48.6 (18.7–76.7)	4 (9.5)
Wong, 2021 [53]	1	0:1 (0:100)	33 (N/A)	0
**Total**	**142**	**34:59 (36.6:63.4)**	**48.0 (35.0–82.0) ^1^**	**7 (6.7)**

^1^ Results from Thompson et al. [10] were not included in synthesis as they were not individually given.

**Table 3:** Conducted diagnostics and the rate of positivity for patients with occult insulinomas extracted from studies.

Study	MRI Conducted *n* (%)	CT Conducted *n* (%)	SRI Conducted *n* (%)	SRI Positive *n* (%)	GLP-1R PET/CT Conducted *n* (%)	GLP-1R PET/CT Positive *n* (%)	EUS Conducted *n* (%)	EUS Positive *n* (%)	SACST Conducted *n* (%)	SACST Positive *n* (%)
Hamburg cohort	2 (100)	2 (100)	1 (50.0)	0	2 (100)	1 (50.0)	2 (100)	0 ^1^	0	-
Antwi, 2015 [26]	3 (75.0)	1 (25.0)	0	-	4 (100)	4 (100)	3 (75)	0	2 (50.0)	0
Antwi, 2018 [12]	25 (73.5)	16 (47.1)	10 (29.4)	1 (2.9)	34 (100)	28 (82.4)	22 (64.7)	7 (31.8)	10 (29.4)	3 (30)
Arjunan, 2024 [27]	1 (100)	1 (100)	1 (100)	0	1 (100)	1 (100)	1 (100)	0	0	-
Bongetti, 2018 [28]	0	1 (100)	1 (100)	0	1 (100)	0 ^2^	1 (100)	1 (100)	1 (100)	1 (100)
Breuer, 2016 [29]	1 (100)	1 (100)	0	-	1 (100)	1 (100)	1 (100)	1 (100)	0	-
Cuthbertson, 2016 [30]	1 (100)	1 (100)	1 (100)	0	1 (100)	1 (100)	1 (100)	0	0	-
Erichsen, 2020 [31]	0 ^2^	0	1 (100)	0 ^3^	1 (100)	0	1 (100)	0	0	-
Guo, 2025 [34]	0	1 (100)	0	-	0	-	1 (100)	1 (100)	0	-
Hofland, 2024 [35]	0	1 (100)	1 (100)	0	1 (100)	1 (100)	1 (100)	1 (100)	0	-
Larose, 2020 [36]	0	1 (100)	1 (100)	1 (100)	0	-	0	-	0	-
Luo, 2015 [40]	1 (100)	1 (100)	1 (100)	0	1 (100)	1 (100)	1 (100)	0	0	-
Luo, 2016 [38]	1 (100)	1 (100)	1 (100)	0	1 (100)	1 (100)	1 (100)	0	1 (100)	0
Mishra, 2022 [41]	N/A	N/A	N/A	N/A	N/A	N/A	N/A	N/A	N/A	N/A
Mohamed Shah, 2021 [42]	1 (100)	1 (100)	0	-	0	-	1 (100)	0	1 (100)	1 (100)
Morera, 2016 [9]	10 (90.9)	9 (81.8)	0	-	0	-	7 (63.6)	5 (71.4)	10 (90.9)	7 (70.0)
Perkov, 2016 [45]	N/A	2 (100)	2 (100)	0	0	-	1 (50.0)	0	2 (100)	2 (100)
Ranaweerage, 2022 [46]	1 (100)	1 (100)	0	-	0	-	0	-	1 (100)	1 (100)
Rashid, 2020 [47]	1 (100)	1 (100)	0	-	0	-	0	-	1 (100)	1 (100)
Sakaki, 2025 [48]	1 (100)	1 (100) ^3^	1 (100)	0	1 (100)	1 (100)	1 (100)	0 ^2^	1 (100)	0
Senica, 2020 [49]	3 (60.0)	5 (100)	5 (100)	0	5 (100)	5 (100)	4 (80.0)	1 (25.0)	0	-
Sowa-Staszczak, 2016 [50]	22 (100)	22 (100)	0	-	22 (100)	22 (100)	0	-	0	-
Subasinghe, 2020 [51]	1 (100)	1 (100)	0	-	0	-	0	-	1 (100)	1 (100)
Thompson, 2015 [10]	42 (100)	N/A	N/A	N/A	N/A	N/A	N/A	N/A	42 (100)	40 (95.2)
Wong, 2021 [53]	1 (100)	1 (100)	0	-	0	-	0	-	1 (100)	1 (100)
**Total**	**118/136 (86.8)**	**72/138 (52.2)**	**27/96 (28.1)**	**2/27** **(7.4)**	**76/96 (79.2)**	**67/76** **(88.2)**	**50/96 (52.1)**	**17/50 (34.0)**	**74/138 (53.6)**	**58/74 (78.4)**

^1^ Initially negative, positive upon repetition for one patient; ^2^ initially negative, retrospectively identified; ^3^ initially negative, positive upon repetition. CT: computed tomography, EUS: endoscopic ultrasound, GLP-1R PET/CT: Glucagon-Like Peptide-1 Receptor positron emission tomography, MRI: magnetic resonance imaging, SACST: selective pancreatic angiography and calcium stimulation catheterisation, SRI: somatostatin receptor imaging.

**Table 4:** Operative details of patients with occult insulinomas.

Study	Number of Operations *n*/All	Minimally Invasive Approach (%) *n* (%)	Intraoperative Ultrasound Conducted *n* (%)	Type of Operation *n* (%)	Postoperative Major Complications ^1^ *n* (%)
Hamburg cohort	2/2	2 (100)	1 (50.0)	Enucleation: 0 PHR: 0 DP: 2 (100) Other: 0	0/2 (0)
Antwi, 2015 [26]	3/4	N/A	N/A	Enucleation: 1 (33.3) PHR: 0 DP: 2 (66.7) Other: 0	N/A
Antwi, 2018 [12]	23/34	N/A	N/A	Enucleation:11 (47.8) PHR: 0 DP: 10 (43.5) Other: 2 (8.7)	N/A
Arjunan, 2024 [27]	1/1	0	1 (100)	Enucleation: 1 (100) PHR: 0 DP: 0 Other: 0	0/1 (0)
Bongetti, 2018 [28]	1/1	1 (100)	N/A	Enucleation: 1 (100) PHR: 0 DP: 0 Other: 0	0/1 (0)
Breuer, 2016 [29]	1/1	0	1 (100)	Enucleation: 1 (100) PHR: 0 DP: 0 Other: 0	0/1 (0)
Cuthbertson, 2016 [30]	1/1	0	N/A	Enucleation: 0 PHR: 0 DP: 0 Other: 1 (100)	0/1 (0)
Erichsen, 2020 [31]	1/1	0	N/A	Enucleation: 0 PHR: 0 DP: 1 (100) Other: 0	1/1 (100)
Guo, 2025 [34]	1/1	0	1 (100)	Enucleation: 0 PHR: 1 (100) DP: 0 Other: 0	0/1 (0)
Hofland, 2024 [35]	1/1	1 (100)	N/A	Enucleation: 1 (100) PHR: 0 DP: 0 Other: 0	0/1 (0)
Larose, 2020 [36]	1/1	1 (100)	N/A	Enucleation: 0 PHR: 0 DP: 1 (100) Other: 0	0/1 (0)
Luo, 2015 [40]	1/1	N/A	1 (100)	Enucleation: 0 PHR: 0 DP: 1 (100) Other: 0	0/1 (0)
Luo, 2016 [38]	1/1	N/A	N/A	Enucleation: N/A PHR: N/A DP: N/A Other: N/A	0/1 (0)
Mishra, 2022 [41]	4/4	4 (100)	4 (100)	Enucleation: N/A PHR: N/A DP: N/A Other: N/A	N/A
Mohamed Shah, 2021 [42]	1/1	N/A	1 (100)	Enucleation: 0 PHR: 0 DP: 1 (100) Other: 0	0/1 (0)
Morera, 2016 [9]	10/11	N/A	6 (60.0)	Enucleation: 6 (60.0) PHR: 2 (20.0) DP: 2 (20.0) Other:0	N/A
Perkov, 2016 [45]	2/2	0	2 (100)	Enucleation: N/A PHR: N/A DP: N/A Other: N/A	N/A
Ranaweerage, 2022 [46]	1/1	0	N/A	Enucleation: 0 PHR: 0 DP: 1 (100) Other: 0	0/1 (0)
Rashid, 2020 [47]	1/1	N/A	N/A	Enucleation: 0 PHR: 0 DP: 1 (100) Other: 0	0/1 (0)
Sakaki, 2025 [48]	1/1	1 (100)	N/A	Enucleation: 1 (100) PHR: 0 DP: 0 Other: 0	0/1 (0)
Senica, 2020 [49]	5/5	N/A	N/A	Enucleation: N/A PHR: N/A DP: N/A Other: N/A	0/5 (0)
Sowa-Staszczak, 2016 [50]	19/22	N/A	N/A	Enucleation: N/A PHR: N/A DP: N/A Other: N/A	1/19 (5.3)
Subasinghe, 2020 [51]	1/1	0	1 (100)	Enucleation: 1 (100) PHR: 0 DP: 0 Other: 0	0/1 (0)
Thompson, 2015 [10]	42/42	N/A	34 (81.0)	Enucleation: 23 (54.8) PHR:1 (2.3) DP: 18 (42.9) Other: 0	0/42 (0)
Wong, 2021 [53]	0/1	-	-	Enucleation: - PHR: - DP: - Other: -	-
**Total**	**125/142 (88.0)**	**10/19 (52.6)**	**53/66 (80.3)**	**Enucleation: 47 (50.0)** **PHR: 4 (4.3)** **DP: 40 (42.6)** **Other: 3 (3.2)**	**2/83 (2.4)**

^1^ Defined as Clavien–Dindo ≥ 3a, PHR: pancreatic head resection, DP: distal pancreatectomy.

**Table 5:** Histopathological characteristics of occult insulinomas.

Study	Mean Tumour Size [mm]		Grading			Localisation	
		(SD)	G1	G2	G3	Head	Body/Tail
Hamburg cohort	13.0	(±4.2)	0	2	0	0	2
Antwi, 2015 [26]	9.3	(±1.2)	N/A	N/A	N/A	0	3
Antwi, 2018 [12]	11.8	(±5.0)	16	4	1	6	19
Arjunan, 2024 [27]	15.0	(N/A)	N/A	N/A	N/A	1	0
Bongetti, 2018 [28]	11.0	(N/A)	N/A	N/A	N/A	0	1
Breuer, 2016 [29]	7.0	(N/A)	1	0	0	1	0
Cuthbertson, 2016 [30]	15.0	(N/A)	1	0	0	0	1
Erichsen, 2020 [31]	10.0	(N/A)	N/A	N/A	N/A	0	1
Guo, 2025 [34]	14.0	(N/A)	N/A	N/A	N/A	0	1
Hofland, 2024 [35]	12.0	(N/A)	1	0	0	0	1
Larose, 2020 [36]	19.0	(N/A)	N/A	N/A	N/A	0	1
Luo, 2015 [40]	N/A	(N/A)	1	0	0	0	1
Luo, 2016 [38]	25.0	(N/A)	0	1	0	0	1
Mishra, 2022 [41]	N/A	(N/A)	N/A	N/A	N/A	N/A	N/A
Mohamed Shah, 2021 [42]	10.1	(N/A)	N/A	N/A	N/A	0	1
Morera, 2016 [9]	13.7	(±4.0)	N/A	N/A	N/A	6	4
Perkov, 2016 [45]	18.0	(±1.4)	N/A	N/A	N/A	2	0
Ranaweerage, 2022 [46]	N/A	(N/A)	1	0	0	0	1
Rashid, 2020 [47]	15.0	(N/A)	1	0	0	0	1
Sakaki, 2025 [48]	23.0	(N/A)	1	0	0	0	1
Senica, 2020 [49]	15.4	(±8.4)	2	3	0	1	4
Sowa-Staszczak, 2016 [50]	N/A	(N/A)	18	0	1	N/A	N/A
Subasinghe, 2020 [51]	10.0	(N/A)	1	0	0	0	1
Thompson, 2015 [10]	16.0	(±0.9)	N/A	N/A	N/A	N/A	N/A
Wong, 2021 [53]	N/A	(N/A)	N/A	N/A	N/A	N/A	N/A
**Total**	**14.2**	**±4.5**	**44/56 (78.6%)**	**10/56** **(17.9%)**	**2/56** **(3.6%)**	**17/62** **(27.4%)**	**45/62** **(72.6%)**

mm: millimetre, SD: standard deviation.

## Data Availability

The data presented in this study are available on request from the corresponding author. The data are not publicly available due to data privacy.

## Abbreviations

The following abbreviations are used in this manuscript:

GLP-1R PET/CT	Glucagon-Like Peptide-1 Receptor positron emission tomography
CDC	Clavien–Dindo classification
cm	centimetre
CoE	Center of Excellence
CT	computed tomography
DGE	delayed gastric emptying
DP	distal pancreatectomy
ENETS	European Neuroendocrine Tumor Society
ERAS	Enhanced Recovery after Surgery
EUS	endoscopic ultrasound
FNA	fine needle aspiration
IOUS	intraoperative ultrasound
IQR	interquartile range
ISGPS	International Study Group for Pancreatic Surgery
MDT	multidisciplinary team
MEN	Multiple endocrine neoplasia
MeSH	medical subject heading
mm	millimetre
MRI	magnetic resonance imaging
N/A	not applicable
NET	neuroendocrine tumour
PHR	pancreatic head resection
pNET	pancreatic neuroendocrine tumour
POPF	postoperative pancreatic fistula
PRISMA	Preferred Reporting Items for Systematic Reviews and Meta-analysis
SACST	selective pancreatic angiography and calcium stimulation catheterisation
SD	standard deviation
SRI	somatostatin receptor imaging
